# Prevalence and determinants of developmental delay among children in low- and middle-income countries: a systematic review and meta-analysis

**DOI:** 10.3389/fpubh.2024.1301524

**Published:** 2024-04-02

**Authors:** Tesfaye Wondmagegn, Bekahegn Girma, Yosef Habtemariam

**Affiliations:** ^1^School of Medicine, College of Medicine and Health Science, Dilla University, Dilla, Ethiopia; ^2^Department of Nursing, College of Medicine and Health Science, Dilla University, Dilla, Ethiopia

**Keywords:** developmental delay, determinants, children, low- and middle-income countries, meta-analysis

## Abstract

**Background:**

Developmental delay is a public health problem in low- and middle-income countries. However, there is no summarized evidence in low- and middle-income countries on developmental delay, and primary studies on this issue show varied and inconclusive results. This systematic review and meta-analysis aimed to assess the pooled magnitude of confirmed developmental delay and its determinants among children in low- and middle-income countries.

**Methods:**

We followed the Preferred Reporting Items for Systematic Reviews and Meta-Analysis (PRISMA) guidelines to write this systematic review and meta-analysis. Primary studies were searched from PubMed, PsycINFO, Hinari, Science Direct, African Journal of Online, Web of Science, and Google Scholar databases. The Newcastle–Ottawa Scale, adapted for the cross-sectional studies, was used to assess the quality of the included studies. Heterogeneity and publication bias were assessed by the I^2^ and Eggers tests, respectively. Due to the high heterogeneity, the random effects model was used for analysis. Odds ratios (ORs) with 95% confidence intervals (CIs) were used to show the association between developmental delay and its determinants.

**Results:**

The pooled prevalence of confirmed developmental delay was 18.83, 95% CI (15.53–22.12). In the subgroup analysis, a high prevalence of developmental delay [26.69% (95% CI, 15.78–37.60)] was observed in studies performed in Africa. Maternal education [3.04; 95% CI (2.05, 4.52)] and low birth weight [3.61; 95% CI (1.72, 7.57)] were significant determinants of developmental delay.

**Conclusion:**

The pooled prevalence of developmental delay in low- and middle-income countries was high as compared to that in high-income countries. Maternal education level and weight at birth were significantly associated with developmental delays. Therefore, strategies should be designed to decrease the rate of low birth weight and the number of illiterate mothers living in low- and middle-income countries.

**Systematic review registration:**

PROSPERO, CRD42024513060.

## Background

Early childhood development is the period from conception to the age of 8 years, which is a critical stage in the physical, emotional, and intellectual growth of a person (1, 2). The brain grows more during these years, and it is a time to lay the groundwork for learning, adapting to change, and ultimately succeeding in life (3). It is the fastest and most vulnerable stage for developmental delay (DD) (4).

DD refers to a wide range of impairments. It is classified as motor, adaptive, cognitive, linguistic, and social–emotional. DD is common and might involve a single domain or many domains (5).

In 2010, approximately 250 million children under the age of 5 years were in danger of not reaching their full potential worldwide (6). In low- and middle-income countries (LMICs), easing child hardship may help with the fulfillment of health and poverty reduction objectives. Few studies have looked at population-representative child development and risk factors for the delay, even though the corpus of information on child development in LMIC contexts is rapidly growing (7–9).

DD is a chronic condition that is a direct cause of most morbidities that occur during life (10). The negative effects of childhood DD have been discussed in several literary studies, including the effects on emotion, behavior (11), parent–child relationships (12), educational achievement (13), and the economic impacts on families and society (14).

Several risk factors associated with the increased risk of DDs have been identified, including malnutrition, extreme poverty, chronic infections, low levels of stimulation in the early years, inadequate cognitive stimulation, iodine deficiency, iron deficiency anemia, maternal depression, and exposure to violence (15).

However, there is no summarized evidence in LMICs on DD, and primary studies on this issue had varied results. Therefore, summarized data are needed to assist in reaching Sustainable Development Goal Target 4.2, which asks countries to ensure that all children have access to high-quality early childhood development care and pre-primary education (16). Therefore, this study aimed to assess the pooled magnitude of DD and its determinants among children in LMICs.

### Research questions

- What is the pooled magnitude of DD among children in LMICs?
- What are the determinants of DD among children in LMICs?

## Methods

Our systematic review and meta-analysis were registered with the registration number CRD42024513060.

### Eligibility criteria

We have used the population intervention comparator outcome (PICO) criteria to describe our research question. However, we had no intervention or comparators because our study was conducted by incorporating cross-sectional studies.

*Population*: Articles conducted among children under 18 years were incorporated.
*Outcome*: The outcome was a confirmed DD.
*Study settings*: Studies conducted in LMICs after 2010 were taken into consideration.
*Study design*: We considered observational studies (cross-sectional, case–control, and cohort) that show the prevalence of DD and/or determinants among children.
*Language*: We considered articles published in English.
*Publication status*: This review considered only published articles.

Studies that do not report the outcome variable and were conducted in the more vulnerable groups (malnutrition) were excluded.

### Information source

PubMed, PsycINFO, Hinari, Science Direct, African Journal of Online (AJOL), Web of Science, Google Scholar databases, and Google were checked for primary articles conducted on DD.

### Search strategy

We used the Preferred Reporting Items for Systematic Reviews and Meta-Analysis (PRISMA) guidelines while producing this review and meta-analysis (17). We utilized “prevalence OR magnitude OR burden AND global developmental delay OR developmental delay OR neurodevelopmental delay AND children AND LMICs” to search primary articles for objective one. “Determinants OR predictors OR associated factors OR risk factors AND developmental delay OR confirmed developmental delay AND children AND LMICs” were used to search articles for objective two (Supplementary material S1). Studies conducted from 2010 until present were incorporated to produce solid proof. The Endnote version X6 program was used to organize citations and check for article duplication.

### Risk of bias assessment

The Ottawa–Newcastle Scale, adapted for the cross-sectional study, was used to evaluate the strengths of the included studies (18). TW and BG independently evaluated the studies with the aforementioned tool. When assessing studies, selection criteria, comparability, and the method used to determine study outcomes were considered. Our review and meta-analysis included studies that scored at least 6 out of 10 on the Ottawa–Newcastle Scale.

### Effect measures

In this review and meta-analysis, we evaluated two objectives. The pooled prevalence of DD among children was calculated by dividing the number of children with DD by the total number of children included in this review and meta-analysis, multiplied by 100. The second objective was to assess the determinants of DD among children in LMICs. In this review and meta-analysis, factors identified as determinants of DD in at least two studies were considered for meta-analysis. We used the odds ratio (OR) to express the pooled effect.

### Selection of studies

Based on the predetermined inclusion and exclusion criteria, TW and BG separately evaluated the eligibility of studies in an unblended and undistinguishable manner. Any discrepancies that arose during the selection of the research were resolved through dialog, either by taking the average results of the two evaluators or by adding a third author.

### Data extraction

Independently, TW and BG extracted all the required data using a regular Microsoft Excel spreadsheet. We used two data extraction formats. The author’s name, publication year, country, study design, sampling technique, sample size, response rate, quality score, and prevalence of DD were the main data extraction arrangements that were organized for the pooled prevalence of DD. Author name, publication year, and frequencies (a, b, c, and d) were assessed to identify determinants.

### Synthesis methods

In this study, heterogeneity was assessed using the I^2^ test and categorized as low, moderate, or high heterogeneity if it was 50%, 50–75%, or > 75%, respectively (19). Analysis was performed using STATA version 16. Because of the high heterogeneity, a random effects model was selected for analysis.

For each original article, the standard error was calculated using the binomial distribution formula. In order to identify the cause of heterogeneity, subgroup analysis was carried out (20–22). To describe the results of this study, systematic review and meta-analysis texts, tables, and forest plots were used.

### Reporting bias assessments

Egger’s and Begg’s statistical tests (23) and funnel plots (24) were used to check publication bias. The presence of evidence for publication bias was declared when the *p*-value was less than 0.05. An odds ratio with a 95% confidence interval (CI) was used to express association.

## Result

### Study search and selection

**Figure 1:**
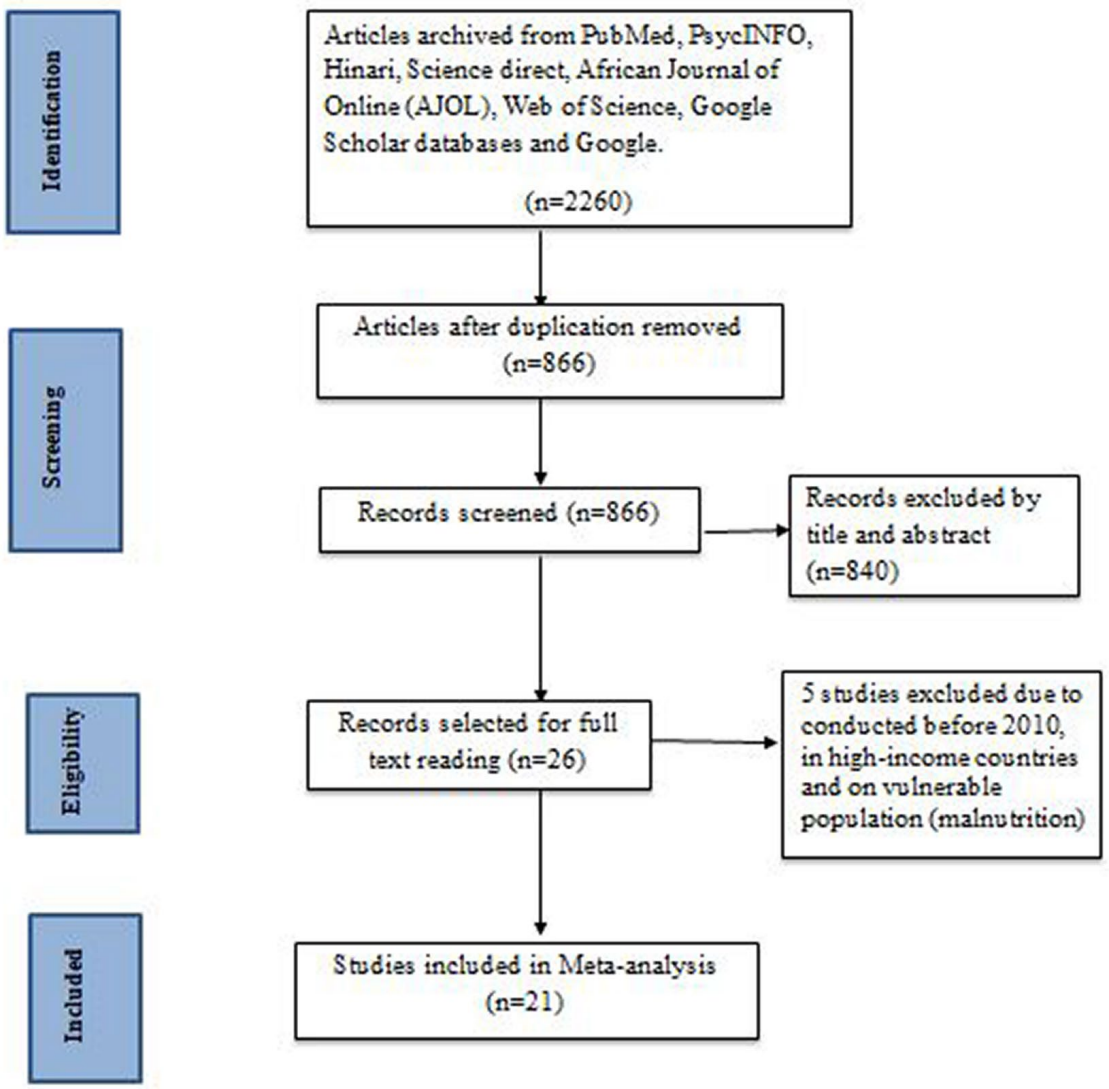
Flow diagram of the studies included in the review of developmental delay among children in Ethiopia, 2023.

We searched full-text publications and human studies published from 2010 until present. A total of 2,260 primary articles were scrutinized from PubMed, PsycINFO, Hinari, Science Direct, AJOL, Web of Science, Google Scholar, and Google databases. Among the total articles, 1,394 and 840 articles were excluded due to duplication and by title and abstract, respectively. Only 26 studies were selected for a full reading. However, an additional five studies were excluded due to them being conducted before 2010, in high-income countries, and on children with malnutrition (25–29) (Table 1). Finally, a total of 21 articles that fulfill the inclusion criteria were selected for the meta-analysis (Figure 1).

**Table 1:** Studies excluded from meta-analysis, 2023.

No	Authors	Country	Reason of exclusion
1	Chi DL et al., 2013	United States	Steered in a high-income country
2	Sitaresmi MN et al., 2008	Indonesia	Piloted before 2010
3	Wei Q et al., 2015	China	Accompanied in a high-income country
4	Zhang J et al., 2018	China	Navigated in a high-income country
5	Saleem J etal., 2021	Pakistan	Conducted among vulnerable children (malnutrition)

### Characteristics of the included articles

This study was conducted on 54,067 children. A total of 21 studies were included in this study. All of the included studies were cross-sectional in design (30–51). Moreover, almost half of the studies used the Ages and Stages Questionnaire (ASQ) to assess DD (27, 30, 31, 36, 37, 39–41, 44, 46, 48). The highest prevalence of DD (56.4%) (43) was reported by a study conducted in Nepal, and the lowest prevalence was in a study conducted in Turkey (6.4%) (41). More than half (12) of the studies were conducted in Asian countries (30, 32, 38, 40, 41, 43, 45–47, 49, 50). More than half (13) of the included studies used probability sampling techniques (simple, systematic, and stratified random sampling) to select participants. Moreover, 17 (81%) of the included studies were conducted among children under 5 years. Finally, among the included studies, only two of them were conducted on children above the age of 6 years (Table 2).

**Table 2:** Characteristics of the included studies to assess the prevalence of developmental delay and its determinants among children in LMICs, 2023 (*n* = 21).

Author	Publication year	Country	Design	Sample size	Prevalence	Tool	Quality score	Sampling technique	Population
Correia L et al.	2019	Brazil	Cross-sectional	3,566	9.2	ASQ	9	Simple	<6 Years
Bhattacharya T et al.	2017	India	Cross-sectional	280	7.9	TDSC	8	Simple	<2 years
Metwally A et al.	2022	Egypt	Cross-sectional	41,640	6.7	VABSA	9	Survey	<12 year
Taye A et al.	2022	Ethiopia	Cross-sectional	390	22.6	ASQ	9	Systematic	<5 years
Gupta S et al.	2021	India	Cross-sectional	240	6.6	TDSC	8	Simple	<5 years
Ali S et al.	2011	India	Cross-sectional	530	19.8	ASQ	7	Simple	<3 years
Sharma N et al.	2019	India	Cross-sectional	450	16.2	RBSK	7	Systematic	<6 years
Murphy R et al.	2019	Malawi	Cross-sectional	960	11.7	MDAT	8	Simple	<10 years
Westgard C et al.	2017	Peru	Cross-sectional	611	26.7	ASQ	9	Survey	<4 years
Dagvadorj A et al.	2018	Mongolia	Cross-sectional	150	11	MORBAS	8	Survey	<2 years
Bello A et al.	2013	Ghana	Cross-sectional	389	44.6	ASQ	9	Survey	<5 years
Shaahmadi F et al.	2014	Iran	Cross-sectional	210	8.6	ASQ	8	Survey	4 month-1 year
Demirci A et al.	2015	Turkey	Cross-sectional	1,514	6.4	ASQ	7	Systematic	<5 years
Miller A et al.	2020	Madagascar	Cross-sectional	432	16	ECDI	8	Simple	3–4 Years
BishwokarmaI A et al.	2022	Nepal	Cross-sectional	165	56.4	DMC	9	Simple	<5 years
Ahishakiye A et al.	2019	Rwanda	Cross-sectional	445	52.6	ASQ	9	Survey	2–3 years
Butchon R et al.	2017	Thailand	Cross-sectional	70	22.9	Denver II	7	Survey	1–5 Years
Yaghini O et al.	2015	Iran	Cross-sectional	680	11.8	ASQ	8	Survey	<5 years
Gunardi H et al.	2019	Indonesia	Cross-sectional	290	10	KPSP	9	Stratified	<3 years
Adeniyi Y et al.	2022	Nigeria	Cross-sectional	587	33.7	ASQ	8	Systematic	6 weeks - 1 year
Sachdeva S et al.	2010	India	Cross-sectional	468	7	ICMR	9	Systematic	<3 years

### Results of syntheses and reporting bias

**Figure 2:**
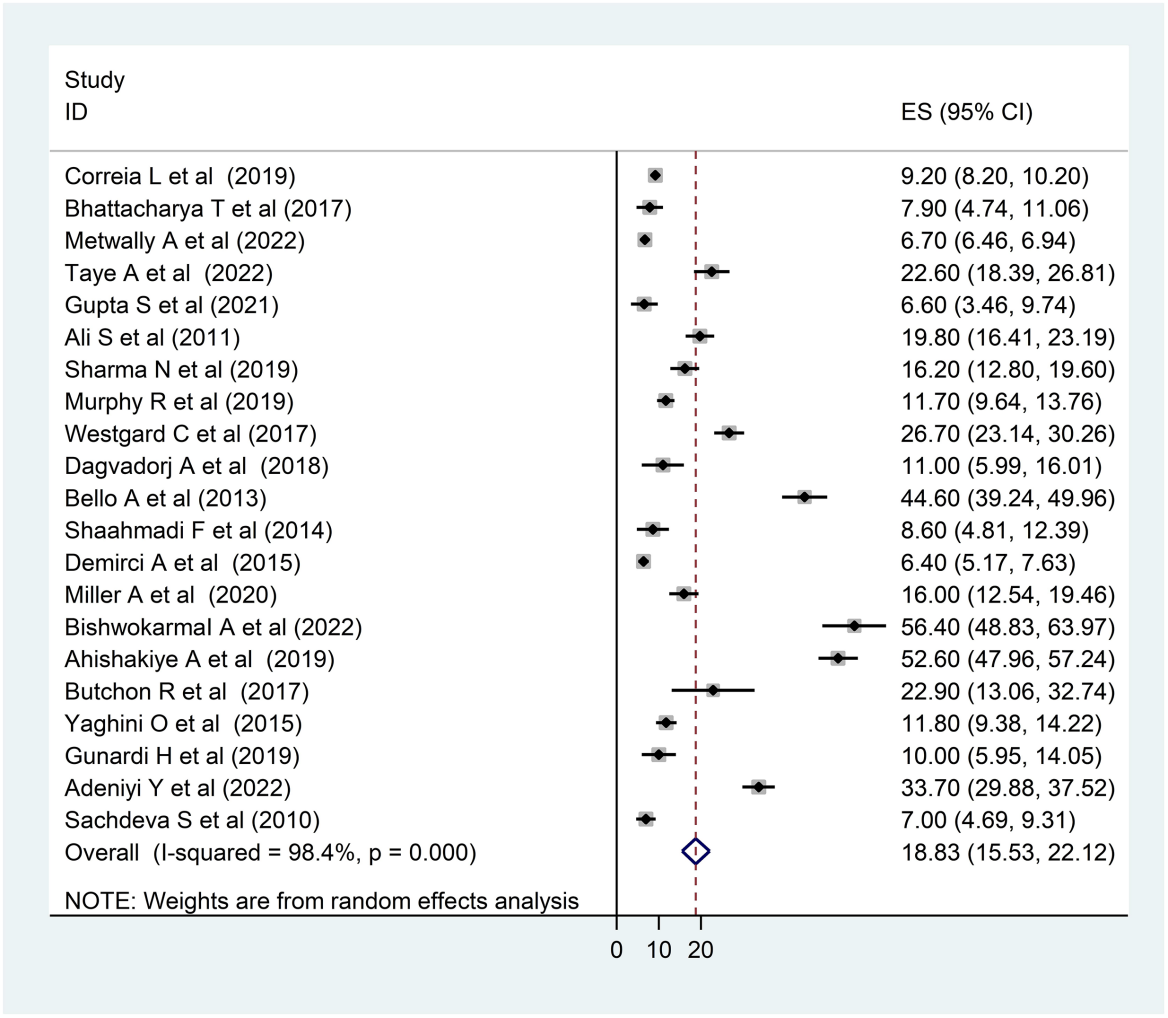
Forest plot of the included studies to assess the pooled prevalence of developmental delay among children in LMICs, 2023 (*n* = 21).

A forest plot was created, as observed in Figure 2, to display the outcomes of the included studies. This systematic review and meta-analysis comprised 21 primary studies to estimate the pooled prevalence of DD. In the present systematic review and meta-analysis, the pooled prevalence of DD in LMICs was 18.83%, with a 95% CI of 15.53–22.12.

**Figure 3:**
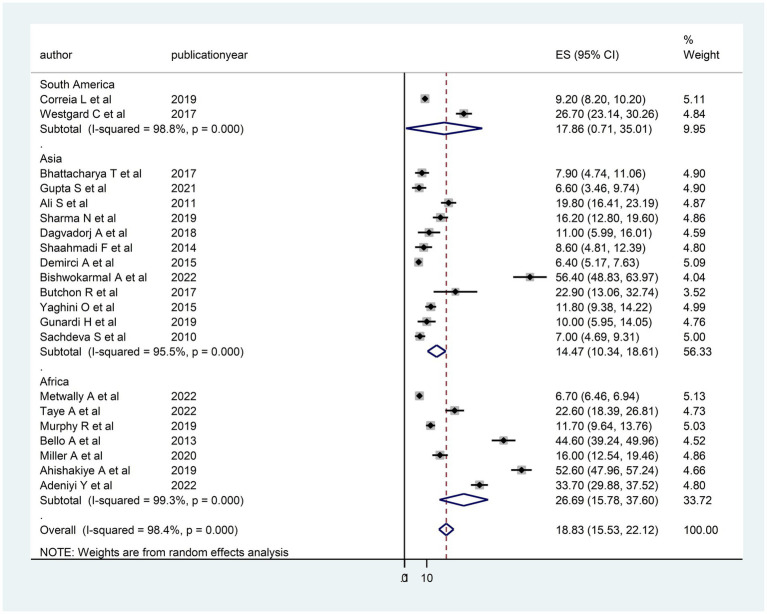
Subgroup analysis (based on region) of the included studies to assess the source of heterogeneity among studies conducted in LMICs, 2023 (*n* = 21).

**Figure 4:**
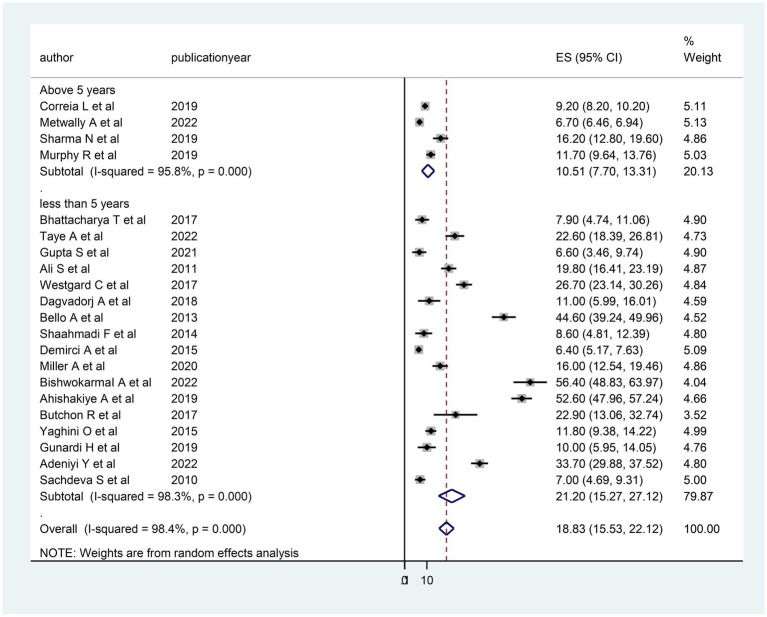
Subgroup analysis (based on the child age) of the included studies to assess the source of heterogeneity among studies conducted in LMICs, 2023 (*n* = 21).

The heterogeneity between studies was high, I^2^ = 98.4%, with a *p*-value of <0.01. To assess the source of heterogeneity, a subgroup analysis was conducted based on population and age. Studies conducted in Africa had a high prevalence of DD [26.69; 95% CI (15.78–37.60)] and heterogeneity (99.3% with *p* < 0.01) as compared to studies conducted in Asian and South American countries (Figure 3). Moreover, a subgroup analysis was conducted based on age, and there was a high prevalence of DD [21.20; 95% CI (15.27–27.12)] and heterogeneity (98.3%; *p* < 0.01) among under children 5 years (Figure 4).

**Figure 5:**
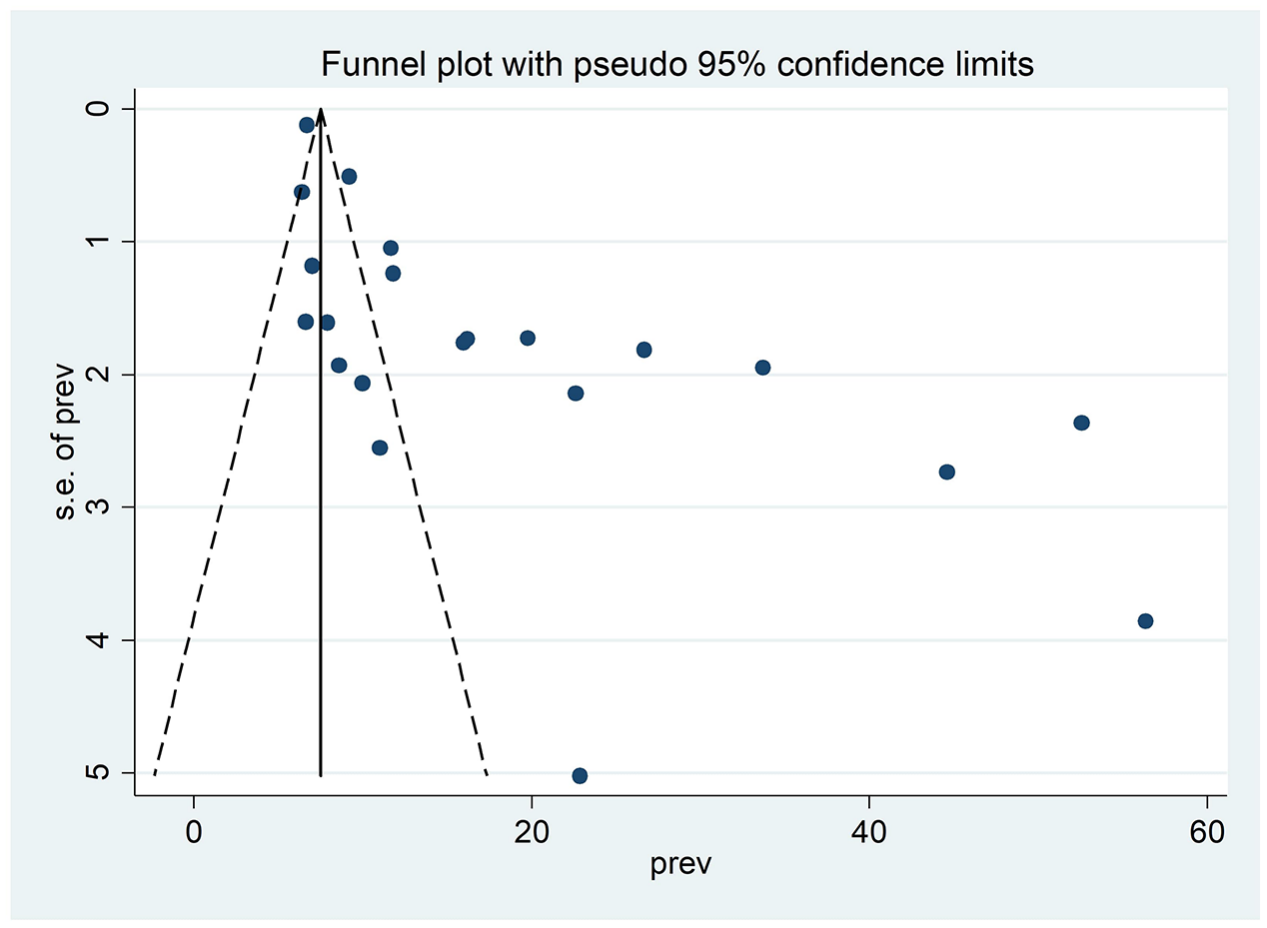
Funnel plot of the included studies to assess publication bias among studies, 2023 (*n* = 21).

Furthermore, in this study, there was publication bias, which was verified with an asymmetric funnel plot (Figure 5) and Egger’s test <0.01. After outlier studies (32, 41, 43, 44) abridged from the analysis, there was no change in Egger’s test value, with a *p*-value of <0.01 and the pooled prevalence was 16.56; 95% CI (13.25–19.67). Therefore, there was evidence for publication bias.

### Determinants of developmental delay

In the present systematic review and meta-analysis, four determinants—birth interval (32, 37), birth weight (32, 44, 50), sex of the child (38, 50), and maternal education (32, 47, 49, 50)—that were reported as determinants in at least two primary studies were selected for meta-analysis. However, only two determinants, birth weight and maternal education levels, had remained significant determinants of DD.

**Figure 6:**
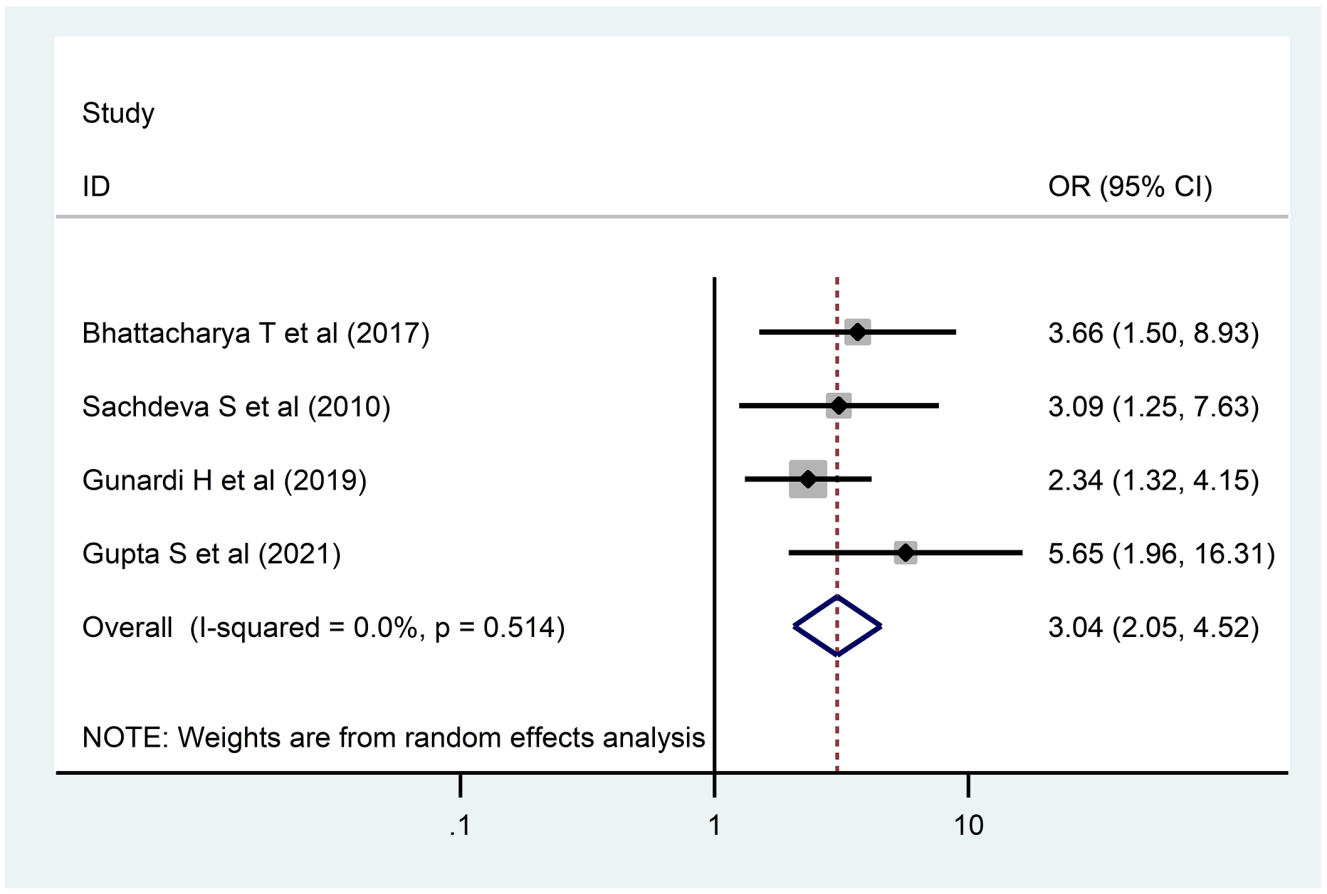
Maternal education and developmental delay among children in LMICs, 2023 (*n* = 4).

**Figure 7:**
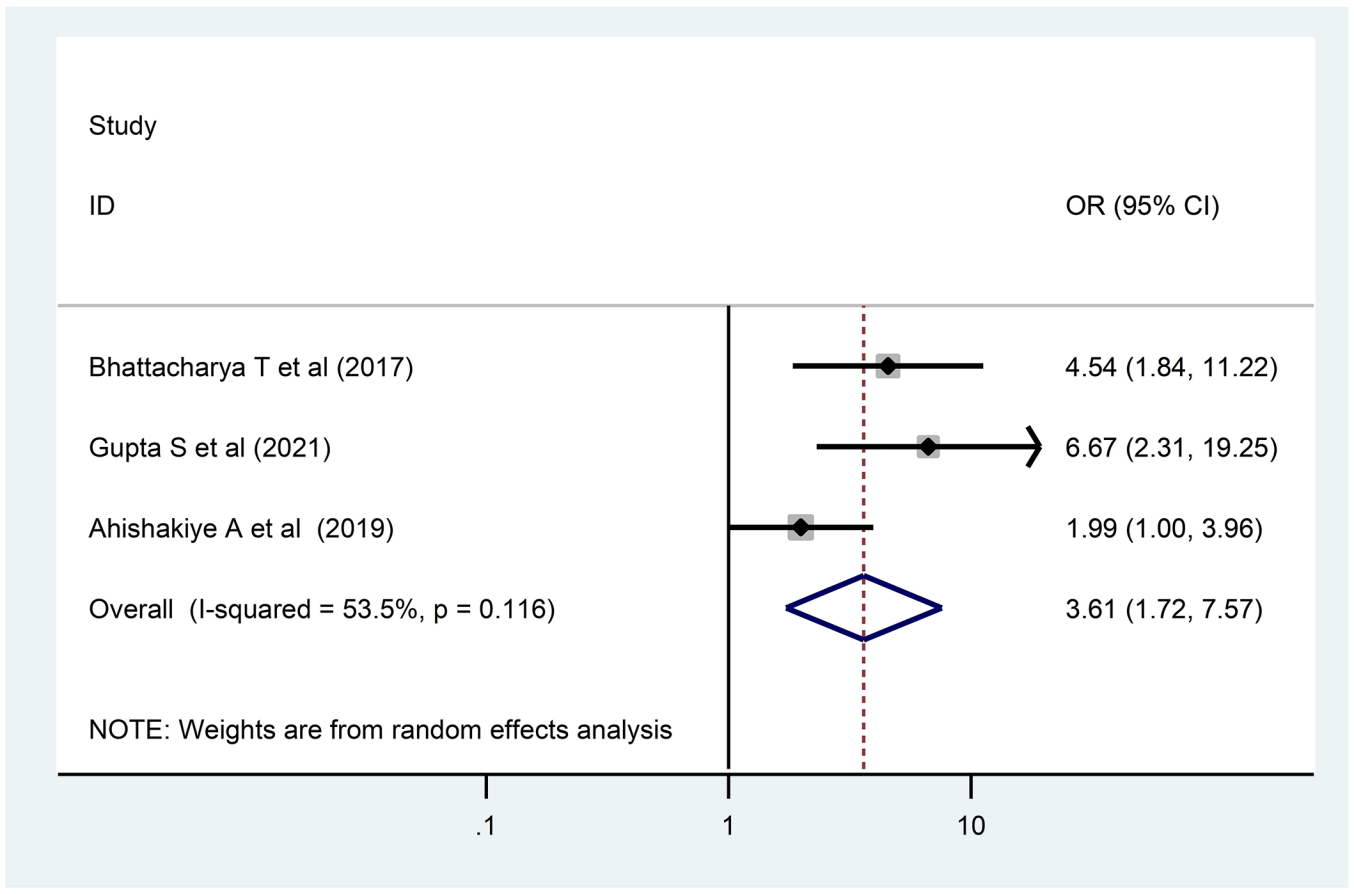
Birth weight and developmental delay among children in LMICs, 2023 (*n* = 3).

A child born to a less educated mother had three times more risk [3.04; 95% CI (2.05, 4.52)] for DD as compared to their comparison group (Figure 6). In addition, children who had low weight at birth had a 3.6 times [3.61; 95% CI (1.72, 7.57)] higher risk for DD as compared to children who had normal birth weight (Figure 7).

## Discussion

This systematic review and meta-analysis aimed to assess the pooled prevalence of DD among children in LMICs. In this review, the pooled prevalence of DD was 18.83%, with a 95% CI of 15.53–22.12.

This finding was low as compared to other studies conducted in LMICs (25%) (52) and China [35.7% (28) and 39.7% (27)], which might be due to the previous study conducted in LMICs estimating the prevalence of suspected DD rather than confirmed DD. Furthermore, this finding might be due to the studies conducted in China assessing people living in poverty-stricken rural areas that have high risk.

Our finding was high as compared to a systematic review and meta-analysis conducted in Iran (14.6%) (53). This difference might be due to the review conducted in Iran only incorporating six primary articles and navigating in a similar study environment with a similar population.

In this study, there was high heterogeneity between studies and publication bias, which might be due to the included studies assessing the outcome variable using different tools, and this study was conducted on a diversified population with different ages, lifestyles, and economic statuses.

In our subgroup analysis, a high prevalence of DD was observed among studies conducted in Africa. This finding was also supported by another study conducted in LMICs (52), which might be due to the fact that most of the African population is living in poverty and that the incidence of malnutrition, inappropriate childcare, and child abuse is high.

In this review and meta-analysis, children who had low weight at birth had high odds of DD, which might be because low birth weight has a latent effect on cognitive, motor, and communication skills (54). Furthermore, a child born to less educated mothers had a high risk of DD. This finding was supported by other studies performed in LMICs (55). This might be due to the fact that educated mothers have good knowledge about child development, which helps them provide quality care for their children.

Even though this was a systematic review and meta-analysis, it had its limitations. The first limitation was the presence of high heterogeneity and publication bias. Second, only studies published in the English language were included. Third, this review and meta-analysis incorporated studies that were assessed using different tools and conducted in different areas and age categories. Finally, studies conducted among preterm neonates were also included.

## Conclusion

The pooled prevalence of DD in LMICs was high, especially in Africa as compared to high-income countries. Low birth weight and low maternal education were found to be strongly associated with DD in the current review and meta-analysis.

## Data availability statement

The original contributions presented in the study are included in the article/Supplementary material, further inquiries can be directed to the corresponding author.

## Author contributions

TW: Conceptualization, Data curation, Formal analysis, Funding acquisition, Investigation, Methodology, Project administration, Resources, Software, Supervision, Validation, Visualization, Writing – original draft, Writing – review & editing. BG: Conceptualization, Data curation, Formal analysis, Funding acquisition, Investigation, Methodology, Project administration, Resources, Software, Supervision, Validation, Visualization, Writing – original draft, Writing – review & editing. YH: Data curation, Methodology, Software, Supervision, Validation, Visualization, Writing – review & editing.

## References

[1] Organization WH. Care for child development: Improving the care of young children. Geneva: WHO (2012).

[2] Organization WH. World report on disability 2011. Geneva: World Health Organization (2011). 26131540

[3] Black MM Walker SP Fernald LC Andersen CT DiGirolamo AM Lu C . Early childhood development coming of age: science through the life course. Lancet. (2017) 389:77–90. doi: 10.1016/S0140-6736(16)31389-7, PMID: 27717614 PMC5884058

[4] Grantham-McGregor S Cheung YB Cueto S Glewwe P Richter L Strupp B. Developmental potential in the first 5 years for children in developing countries. Lancet. (2007) 369:60–70. doi: 10.1016/S0140-6736(07)60032-4, PMID: 17208643 PMC2270351

[5] Brown KA Parikh S Patel DR. Understanding basic concepts of developmental diagnosis in children. Transl Pediatr. (2020) 9:S9–S22. doi: 10.21037/tp.2019.11.04, PMID: 32206580 PMC7082247

[6] Lu C Black MM Richter LM. Risk of poor development in young children in low-income and middle-income countries: an estimation and analysis at the global, regional, and country level. Lancet Glob Health. (2016) 4:e916–22. doi: 10.1016/S2214-109X(16)30266-2, PMID: 27717632 PMC5881401

[7] Valla L Wentzel-Larsen T Hofoss D Slinning K. Prevalence of suspected developmental delays in early infancy: results from a regional population-based longitudinal study. BMC Pediatr. (2015) 15:1–8. doi: 10.1186/s12887-015-0528-z 26678149 PMC4683867

[8] Bailey S Boddy K Briscoe S Morris C. Involving disabled children and young people as partners in research: a systematic review. Child Care Health Dev. (2015) 41:505–14. doi: 10.1111/cch.12197, PMID: 25323964

[9] Sajedi F Vameghi R Kraskian Mujembari A. Prevalence of undetected developmental delays in I ranian children. Child Care Health Dev. (2014) 40:379–88. doi: 10.1111/cch.12042 23461377

[10] Mathers CD. History of global burden of disease assessment at the World Health Organization. Arch Public Health. (2020) 78:1–13. doi: 10.1186/s13690-020-00458-3 32850124 PMC7443850

[11] Emerson E Einfeld S. Emotional and behavioural difficulties in young children with and without developmental delay: a bi-national perspective. J Child Psychol Psychiatry. (2010) 51:583–93. doi: 10.1111/j.1469-7610.2009.02179.x, PMID: 20015191

[12] Baker BL McIntyre LL Blacher J Crnic K Edelbrock C Low C. Pre-school children with and without developmental delay: behaviour problems and parenting stress over time. J Intellect Disabil Res. (2003) 47:217–30. doi: 10.1046/j.1365-2788.2003.00484.x, PMID: 12787154

[13] Dornelas LF Magalhães LC. Functional performance of school children diagnosed with developmental delay up to two years of age. Rev Paul Pediatr. (2016) 34:78–85. doi: 10.1016/j.rpped.2015.05.004, PMID: 26553573 PMC4795725

[14] Quach J Oberklaid F Gold L Lucas N Mensah FK Wake M. Primary health-care costs associated with special health care needs up to age 7 years: a ustralian population-based study. J Paediatr Child Health. (2014) 50:768–74. doi: 10.1111/jpc.12649, PMID: 24923806

[15] Walker SP Wachs TD Gardner JM Lozoff B Wasserman GA Pollitt E . Child development: risk factors for adverse outcomes in developing countries. Lancet. (2007) 369:145–57. doi: 10.1016/S0140-6736(07)60076-2 17223478

[16] Griggs D Stafford-Smith M Gaffney O Rockström J Öhman MC Shyamsundar P . Sustainable development goals for people and planet. Nature. (2013) 495:305–7. doi: 10.1038/495305a 23518546

[17] Page MJ Moher D Bossuyt PM Boutron I Hoffmann TC Mulrow CD . PRISMA 2020 explanation and elaboration: updated guidance and exemplars for reporting systematic reviews. BMJ. (2021) 372:n160. doi: 10.1136/bmj.n160 33781993 PMC8005925

[18] Peterson J Welch V Losos M Tugwell P. The Newcastle-Ottawa scale (NOS) for assessing the quality of nonrandomised studies in meta-analyses. Ottawa: Ottawa Hospital Research Institute (2011).

[19] Higgins JP Thompson SG Deeks JJ Altman DG. Measuring inconsistency in meta-analyses. BMJ. (2003) 327:557–60. doi: 10.1136/bmj.327.7414.557, PMID: 12958120 PMC192859

[20] Borenstein M Higgins JP. Meta-analysis and subgroups. Prev Sci. (2013) 14:134–43. doi: 10.1007/s11121-013-0377-7, PMID: 23479191

[21] Marušić MF Fidahić M Cepeha CM Farcaș LG Tseke A Puljak L. Methodological tools and sensitivity analysis for assessing quality or risk of bias used in systematic reviews published in the high-impact anesthesiology journals. BMC Med Res Methodol. (2020) 20:1–10. doi: 10.1186/s12874-020-00966-4 PMC7236513 32423382

[22] Patsopoulos NA Evangelou E Ioannidis JP. Sensitivity of between-study heterogeneity in meta-analysis: proposed metrics and empirical evaluation. Int J Epidemiol. (2008) 37:1148–57. doi: 10.1093/ije/dyn065, PMID: 18424475 PMC6281381

[23] Egger M Smith GD Schneider M Minder C. Bias in meta-analysis detected by a simple, graphical test. BMJ. (1997) 315:629–34. doi: 10.1136/bmj.315.7109.629, PMID: 9310563 PMC2127453

[24] Sterne JA Egger M. Funnel plots for detecting bias in meta-analysis: guidelines on choice of axis. J Clin Epidemiol. (2001) 54:1046–55. doi: 10.1016/S0895-4356(01)00377-8 11576817

[25] Chi DL Rossitch KC Beeles EM. Developmental delays and dental caries in low-income preschoolers in the USA: a pilot cross-sectional study and preliminary explanatory model. BMC Oral Health. (2013) 13:1–10. doi: 10.1186/1472-6831-13-53 24119240 PMC3906997

[26] Sitaresmi MN Ismail D Wahab A. Risk factors of developmental delay: a community-based study. Paediatr Indones. (2008) 48:161–5. doi: 10.14238/pi48.3.2008.161-5

[27] Wei Q Zhang J Scherpbier R Zhao C Luo S Wang X . High prevalence of developmental delay among children under three years of age in poverty-stricken areas of China. Public Health. (2015) 129:1610–7. doi: 10.1016/j.puhe.2015.07.036, PMID: 26318615

[28] Zhang J Guo S Li Y Wei Q Zhang C Wang X . Factors influencing developmental delay among young children in poor rural China: a latent variable approach. BMJ Open. (2018) 8:e021628. doi: 10.1136/bmjopen-2018-021628, PMID: 30173158 PMC6120651

[29] Saleem J Zakar R Bukhari GMJ Fatima A Fischer F. Developmental delay and its predictors among children under five years of age with uncomplicated severe acute malnutrition: a cross-sectional study in rural Pakistan. BMC Public Health. (2021) 21:1–10. doi: 10.1186/s12889-021-11445-w 34266406 PMC8281691

[30] Ali SS. Assessment of growth and global developmental delay: a study among young children in a rural community of India. Int Multidiscip Res J. (2011) 1:31–4.

[31] Correia LL Rocha HAL Sudfeld CR Rocha SGMO Leite ÁJM Campos JS . Prevalence and socioeconomic determinants of development delay among children in Ceará, Brazil: a population-based study. PLoS One. (2019) 14:e0215343. doi: 10.1371/journal.pone.0215343, PMID: 31689294 PMC6830766

[32] Gupta S Shrivastava P Samsuzzaman M Banerjee N Das DK. Developmental delay among children under two years of age in slums of Burdwan municipality: a cross-sectional study. J Family Med Prim Care. (2021) 10:1945–9. doi: 10.4103/jfmpc.jfmpc_1926_20, PMID: 34195129 PMC8208187

[33] Khandelwal N Mandliya J Nigam K Patil V Mathur A Pathak A. Determinants of motor, language, cognitive, and global developmental delay in children with complicated severe acute malnutrition at the time of discharge: an observational study from Central India. PLoS One. (2020) 15:e0233949. doi: 10.1371/journal.pone.0233949, PMID: 32479548 PMC7263621

[34] Metwally AM Abdallah AM Salah El-Din EM Khadr Z Raouf ERA Elghareeb NA . A national prevalence and profile of single and multiple developmental delays among children aged from 1 year up to 12 years: an Egyptian community-based study. Child Adolesc Psychiatry Ment Health. (2022) 16:63. doi: 10.1186/s13034-022-00498-3, PMID: 35932037 PMC9356393

[35] Murphy R Jolley E Lynch P Mankhwazi M Mbukwa J Bechange S . Estimated prevalence of disability and developmental delay among preschool children in rural Malawi: findings from “Tikule Limodzi,” a cross‐sectional survey. Child Care Health Dev. (2020) 46:187–94. doi: 10.1111/cch.12741, PMID: 31925814 PMC7027747

[36] Westgard C Alnasser Y. Developmental delay in the Amazon: the social determinants and prevalence among rural communities in Peru. PLoS One. (2017) 12:e0186263. doi: 10.1371/journal.pone.0186263, PMID: 29023517 PMC5638337

[37] Wondemagegn AT Mulu A: Effects of Nutritional Status on Neurodevelopment of Children Aged Under Five Years in East Gojjam, Northwest Ethiopia. A community-based study. Int. J. Gen. Med. (2021) 15:5533–45. doi: 10.2147/IJGM.S369408 PMC9189147 35707740

[38] Dagvadorj A Ganbaatar D Balogun O Yonemoto N Bavuusuren B Takehara K . Maternal socio-demographic and psychological predictors for risk of developmental delays among young children in Mongolia. BMC Pediatr. (2018) 18:68–8. doi: 10.1186/s12887-018-1017-y, PMID: 29458342 PMC5817794

[39] Bello AI Quartey JN Appiah LA. Screening for developmental delay among children attending a rural community welfare clinic in Ghana. BMC Pediatr. (2013) 13:1–7. doi: 10.1186/1471-2431-13-119 23937954 PMC3751115

[40] Shaahmadi F Khushemehri G Arefi Z Karimyan A Heidari F. Developmental delay and its effective factors in children aged 4 to12 months. Int J Pediatr. (2015) 3:396–402.

[41] Demirci A Kartal M. The prevalence of developmental delay among children aged 3–60 months in Izmir, Turkey. Child Care Health Dev. (2016) 42:213–9. doi: 10.1111/cch.12289, PMID: 26493366

[42] Miller AC Garchitorena A Rabemananjara F Cordier L Randriamanambintsoa M Rabeza V. Razanadrakoto H-TR, Rakoto Ramakasoa R, RamahefarisonTiana O, Ratsimbazafy BN: factors associated with risk of developmental delay in preschool children in a setting with high rates of malnutrition: a cross-sectional analysis of data from the IHOPE study, Madagascar. BMC Ped. (2020) 20:1–11. doi: 10.1186/s12887-020-1985-6 PMC7059323 32138722

[43] Bishwokarma A Shrestha D Bhujel K Chand N Adhikari L Kaphle M . Developmental delay and its associated factors among children under five years in urban slums of Nepal. PLoS One. (2022) 17:e0263105. doi: 10.1371/journal.pone.0263105, PMID: 35143516 PMC8830665

[44] Ahishakiye A Abimana MC Beck K Miller AC Betancourt TS Magge H . Developmental outcomes of preterm and low birth weight toddlers and term peers in Rwanda. Ann Glob Health. (2019) 85:147. doi: 10.5334/aogh.2629, PMID: 31871910 PMC6923771

[45] Butchon R Liabsuetrakul T. The development and growth of children aged under 5 years in northeastern Thailand: a cross-sectional study. J Child Adolesc Behav. (2017) 5:2. doi: 10.4172/2375-4494.1000334

[46] Yaghini O Kelishadi R Keikha M Niknam N Sadeghi S Najafpour E . Prevalence of developmental delay in apparently normal preschool children in Isfahan, Central Iran. Iran J Child Neurol. (2015) 9:17–23. PMID: 26401149 PMC4577694

[47] Gunardi H Nugraheni RP Yulman AR Soedjatmiko S Sekartini R Medise BE . Growth and developmental delay risk factors among under-five children in an inner-city slum area. Paediatr Indones. (2019) 59:276–83. doi: 10.14238/pi59.5.2019.276-83

[48] Adeniyi Y Asinobi A Idowu O Adelaja A Lagunju I. Early-onset developmental impairments among infants attending the routine immunization clinic at the university college hospital, Ibadan, Nigeria. Int Health. (2022) 14:97–102. doi: 10.1093/inthealth/ihab016, PMID: 33822058 PMC8769952

[49] Sachdeva S Amir A Alam S Khan Z Khalique N Ansari M. Global developmental delay and its determinants among urban infants and toddlers: a cross sectional study. Indian J Pediatr. (2010) 77:975–80. doi: 10.1007/s12098-010-0151-9, PMID: 20734165

[50] Bhattacharya T Ray S Das DK. Developmental delay among children below two years of age: a cross-sectional study in a community development block of Burdwan district, West Bengal. Int J Commun Med Public Health. (2017) 4:1762–7. doi: 10.18203/2394-6040.ijcmph20171798

[51] Sharma N Masood J Singh S Ahmad N Mishra P Singh S . Assessment of risk factors for developmental delays among children in a rural community of North India: a cross-sectional study. J Educ Health Promot. (2019) 8:112. doi: 10.4103/jehp.jehp_405_18 31334264 PMC6615120

[52] Gil JD Ewerling F Ferreira LZ Barros AJ. Early childhood suspected developmental delay in 63 low-and middle-income countries: large within-and between-country inequalities documented using national health surveys. J Glob Health. (2020) 10:010427. doi: 10.7189/jogh.10.010427, PMID: 32566165 PMC7295453

[53] Sajedi F Ahmadi Doulabi M Vameghi R Baghban AA Mazaheri MA Mahmodi Z . Development of children in Iran: a systematic review and meta-analysis. Global J Health Sci. (2016) 8:145–61. doi: 10.5539/gjhs.v8n8p145 PMC5016360 27045395

[54] Hilaire M Andrianou XD Lenglet A Ariti C Charles K Buitenhuis S . Growth and neurodevelopment in low birth weight versus normal birth weight infants from birth to 24 months, born in an obstetric emergency hospital in Haiti, a prospective cohort study. BMC Pediatr. (2021) 21:143. doi: 10.1186/s12887-021-02605-3, PMID: 33761917 PMC7988959

[55] Geldsetzer P Williams TC Kirolos A Mitchell S Ratcliffe LA Kohli-Lynch MK . The recognition of and care seeking behaviour for childhood illness in developing countries: a systematic review. PLoS One. (2014) 9:e93427. doi: 10.1371/journal.pone.0093427, PMID: 24718483 PMC3981715

