# Supplementary material for: Prevalence and determinants of developmental delay among children in low- and middle-income countries: a systematic review and meta-analysis
Source: Front Public Health. 2024 Apr 2;12:1301524. doi: 10.3389/fpubh.2024.1301524 (PMC11018911; doi:10.3389/fpubh.2024.1301524)
Supplement: Supplementary file 1 [file Data_Sheet_1.docx]

**Supplementary file 1: Table S1**. Search strategies for prevalence and determinants of developmental delay among children in low and middle-income countries, 2023: A systematic review and meta-analysis

| **S.N.** | **Database** | **Search strategy** |
| --- | --- | --- |
| 1 | PubMed | (((Prevalence OR Magnitude OR Burden Global developmental delay OR developmental delay OR neurodevelopmental delay) AND children) AND LMICs’. Also  (((Determinants OR predictors OR associated factors OR risk factors AND Developmental delay) AND children) AND LMICs) |
| 2 | Google scholar | developmental delay OR Neuro developmental delay OR global developmental delay and "children" and low and middle income countries |
| 3 | PsycINFO | (developmental delay *AND* **Any Field**:  Children *AND* **Publication Type**: Journal |
| 4 | Scopus | TITLE-ABS-KEY (''developmental delay OR Neuro developmental delay AND Determinants OR Predictors OR factors AND children'' AND low and middle income countries) |
| 5 | Hinari | developmental delay OR neurodevelopmental delay AND determinants OR predictors OR factors AND children AND LMICs’ (full text article AND English language) |
| 6 | Science direct | Find articles with these terms: developmental delay AND Determinants OR Predictors OR factors AND low and middle income countries |
| 7 | Web of sciences | TOPIC: (developmental delay Determinants OR Predictors OR factors AND low and middle income countries) |
| 8 | AJOL | ab(prevalence OR magnitude AND Determinants OR Predictors OR factors AND developmental delay and children and) AND (low and middle income countries) |
